# Factors associated with overweight: are the conclusions influenced by choice of the regression method?

**DOI:** 10.1186/s12889-016-3340-2

**Published:** 2016-07-26

**Authors:** Leidjaira Lopes Juvanhol, Raquel Martins Lana, Renata Cabrelli, Leonardo Soares Bastos, Aline Araújo Nobre, Lúcia Rotenberg, Rosane Härter Griep

**Affiliations:** 1 Escola Nacional de Saúde Pública, Fundação Oswaldo Cruz, Rio de Janeiro, RJ Brazil; 2 Programa de Computação Científica, Fundação Oswaldo Cruz, Rio de Janeiro, RJ Brazil; 3 Instituto Oswaldo Cruz, Fundação Oswaldo Cruz, Rio de Janeiro, RJ Brazil

**Keywords:** Body mass index, Overweight, Regression analysis, Methods, Nurses

## Abstract

**Background:**

Different analytical techniques have been used to study the determinants of overweight. However, certain commonly used techniques may be limited by the continuous nature and skewed distribution of body mass index (BMI) data. In this article, different regression models are compared to identify the best approach for analysing predictors of BMI.

**Methods:**

Data collected on 2270 nurses at 18 public hospitals in Rio de Janeiro, RJ (2010–2011) were analysed (80.6 % of the respondents). The explanatory variables considered were *age*, *marital status*, *race/colour*, *mother’s schooling*, *domestic overload*, *years worked at night*, *consumption of fried food*, *physical inactivity*, *self-rated health* and *BMI at age 20 years*. In addition to gamma regression, regarded as the reference method for selecting the set of explanatory variables described here, other modelling strategies – including linear, quantile (for the 0.25, 0.50 and 0.75 quantiles), binary and multinomial logistic regression – were compared in terms of final results and measures of fit.

**Results:**

The variables *age*, *marital status*, *race/colour*, *domestic overload*, *self-rated health*, *physical inactivity* and *BMI at age 20 years* were significantly associated with BMI, independently of the method used. In the same way, *consumption of fried food* was significant in all the models, but a dose–response pattern was identified only in the gamma and normal models and the quantile model for the 0.75 quantile. *Years worked at night* was also associated with BMI in these three models only. The variable *mother’s schooling* returned significant results only for the category 12 or more years of schooling, except for overweight in the multinomial model and for the 0.50 quantile in the quantile model, in which the two categories were not significant. The results of the quantile regression showed that, generally, the effects of the variables investigated were greater in the upper quantiles of the BMI distribution. Of the models using BMI in its continuous form, the gamma model showed best fit, followed by the quantile models (0.25 and 0.5 quantiles).

**Conclusions:**

The different strategies used produced similar results for the factors associated with BMI, but differed in the magnitude of the associations and goodness of fit. We recommend using the different approaches in combination, because they furnish complementary information on the problem studied.

**Electronic supplementary material:**

The online version of this article (doi:10.1186/s12889-016-3340-2) contains supplementary material, which is available to authorized users.

## Background

Body mass index (BMI) data are commonly modelled by logistic regression (binary and multinomial) [1, 2] and robust Poisson regression [3, 4] for the purpose of identifying factors associated with overweight/obesity. Even though these two approaches yield estimates of measures of association (respectively, odds ratio [OR] and prevalence ratio) that are easier to interpret, they suffer from important limitations. Categorisation of an originally continuous variable entails information loss, because it assumes that individuals in the same stratum are homogeneous. In addition, introducing variability within categories results in loss of statistical power [5].

Another method often used in studies of determinants of nutritional status is linear regression [6, 7]. However, as BMI data are asymmetrically distributed, they must undergo transformations, making results harder to interpret [8]. In that regard, another approach that is quite appropriate, although little used, is gamma regression, which is indicated for modelling skewed and strictly positive continuous variables [9].

All the methods described, however, estimate the mean effect of independent variables, but do not make it possible to evaluate whether that effect is homogeneous and significant across the entire BMI distribution. Recent studies using the quantile regression method have demonstrated that important risk factors, such as lack of schooling, inappropriate diet, physical inactivity and family history of obesity, influence eutrophic and obese individuals differently. The effect of these variables was observed to increase progressively in the upper percentiles of the BMI distribution [10, 11]. Although that approach is quite robust and provides additional information in relation to the usual methods, it is rarely used in epidemiology [12].

In this context, it is extremely important to use methods suited to the study in question, because overweight is one of the six leading risk factors for the global burden of disease and, in 2010, was responsible for some 3.4 million deaths worldwide [13]. It is estimated that more than 2 billion people currently have a BMI of 25 kg/m^2^ or more [14].

Accordingly, given the diversity of methods employed to investigate the relationship between overweight and its potential risk factors, the aim of this study was to compare different regression models on the basis of data collected from nurses at public hospitals in Rio de Janeiro, and to identify the best approach for application in this scenario.

## Methods

### Data

Data on 2818 nurses at 18 public hospitals in the municipality of Rio de Janeiro, RJ, Brazil, were collected between March 2010 and November 2011. The sample used in the analyses comprised 2270 women (80.6 % of the respondents) with complete information for the variables studied. The self-completed questionnaire contained three large blocks of questions, covering characteristics of professional and household work, health and socioeconomic position (see Additional file 1). The study was approved by the Oswaldo Cruz Foundation (Fiocruz) research ethics committee (No. 472/08) and authorised by representatives of the hospitals. All participants signed declarations of informed consent. The study is described in detail in Griep et al. [15].

### Statistical methods

Factors associated with overweight in the literature [6, 16–18] were tested by bivariate analysis with BMI as response variable (Pearson Linear Correlation for continuous variables and *t*-test and ANOVA for variables with two or more categories, respectively). At first, all explanatory variables with p-value ≤ 0.20 in the bivariate analysis were modelled by gamma regression, using the identity link function. That model was chosen in view of the continuous, strictly positive and skewed nature of the dependent variable. We used a backward procedure for variable selection. From the complete model, the variable that contributed least (largest p-value) was removed. This process was repeated until all the remaining variables were significant (p-value ≤ 0.05). Lastly, the model was tested by reintroducing the variables that had been eliminated, but none contributed significantly to the model. At the final gamma model, we have used Generalized Variance Inflation Factor (GVIF) [19] to assessed collinearity between the significant BMI predictors. We found GVIF values adjusted by the degree of freedom very close to unity, indicating that collinearity had no impact on the precision of estimation of coefficients.

The significant BMI predictors at the final gamma model were: *age* (in complete years); *marital status* (with or without partner); *race/colour*, self-declared using Brazil’s population census classification (black, white and brown [or mixed-race] – participants who reported skin colour as yellow [or Asian] and indigenous were excluded from the analyses because of low frequency, *n* = 62 and *n* = 6, respectively); *mother’s schooling* (0-8, 9-11 and 12 or more years of schooling); *domestic overload*, given by the product of the score for responsibility for four basic types of household chore – cleaning, cooking, washing and ironing – and the number of residents in the household, excluding the respondent herself, and dichotomised by the second tercile of the distribution (the upper tercile [high domestic overload] was compared to the two lower ones [low domestic overload]) [20]; *years worked at night* (in years); *consumption of fried food* (never or less than once a month, 1 to 3 times a month, 1 to 3 times a week, 4 to 6 times a week, and daily); *physical inactivity* (yes or no), assessed from the question “In the last two weeks, you practiced some kind of physical activity?”; *self-rated health* (good or poor) and *BMI at age 20 years* (in kg/m^2^).

The explanatory variables tested in the bivariate analysis and/or gamma regression but that were not statistically significant BMI predictors were the following: *education level* (university degree, postgraduate, and Master’s or PhD); *self-reported insomnia symptoms* (yes or no), defined as those who answered often or always to any of the questions concerning “Difficulty in falling asleep”, “Waking up during the night (more than three times)”, or “Waking up before the desired time and not manage to sleep again” [21]; *smoking* (never smoked, ex-smoker, and current smoker); *alcohol consumption* (never, once a month or less, 2 to 4 times a month, 2 to 3 times a week, and 4 or more times a week); *fruits consumption* (never or less than once a month, 1 to 3 times a month, 1 to 3 times a week, 4 to 6 times a week, and daily); *vegetables consumption* (never or less than once a month, 1 to 3 times a month, 1 to 3 times a week, 4 to 6 times a week, and daily); *contraceptive use* (yes or no); *number of biological children* (in units); *presence of children under six years old* (yes or no); and *number of employments* (one, and two or more).

From the selected set of explanatory variables, different regression models [22, 23] were analysed and their final results compared. In addition to gamma regression (described above and used as the reference method), multiple linear regression was performed to model the relationship between these variables and BMI. In order to identify the explanatory variables’ effects on specific quantiles of the BMI distribution, quantile regression models were also constructed for the 0.25, 0.50 and 0.75 quantiles. Graphs were plotted by estimating coefficients for regular 5-quantile intervals, from the 5th to the 95th quantile. Due to sectional nature of this study, is not possible to establish cause and effect relationship. However, to remain consistent with literature about quantile regression, the word “effect” is used here to indicate association, not causation.

Two other models were evaluated using BMI in its categorical form. Binary logistic regression was performed with overweight as the dependent variable (BMI ≥ 25). Multinomial logistic regression contemplated three levels: normal (BMI < 25), overweight (25 ≤ BMI < 30) and obesity (BMI ≥ 30), taking the former as the reference category. To make the results of these two models easier interpretable, they were expressed as the exponential of the regression coefficient, which corresponds to an estimated OR.

The Akaike Information Criterion (AIC) was used to compare gamma, linear [24] and quantile [23] models, and lower values were considered best fitted. The binary and multinomial logistic models could not be compared using the AIC, because they do not model exactly the same response variable.

The approaches were also compared by estimating BMI values (gamma, linear and quantile) or likelihoods (binary and multinomial logistic), by model, for each of three specific profiles of individual. These profiles, here termed healthy, intermediate and unhealthy, were constructed from the results of the analyses of the factors associated with BMI. The healthy profile was obtained from characteristics regarded as protective against overweight, and vice versa for the unhealthy profile. The intermediate profile was identified, in the case of the dichotomous variables, by using the most frequent category or, for polytomous variables with odd numbers of categories, the median stratum. For continuous variables, the mean value was used for the intermediate profile, and the 5th and 95th percentiles of the distribution, for the extreme profiles.

In that way, three profiles were formed with the following characteristics: healthy (26 years old, no partner, white, mother with 0-8 years’ schooling, low domestic overload, never worked nights, good self-rated health, consumes fried food less than once a month, physically active and BMI at age 20 years 16.9 kg/m^2^); intermediate (39.5 years old, with partner, brown, mother with 9-11 years’ schooling, low domestic overload, 7.1 years worked at night, good self-rated health, consumes fried food from one to three times a week, physically inactive and BMI at age 20 years 21.2 kg/m^2^); and unhealthy (56 years old, with partner, black, mother with 12 or more years’ schooling, high domestic overload, 21 years worked at night, poor self-rated health, consumes fried food daily, physically inactive and BMI at age 20 years 26.7 kg/m^2^).

The models were also evaluated, where possible, by residual analysis and measures of goodness of fit (Hosmer-Lemeshow test for the binary logistic model, and the deviance function for the rest). All analyses were performed using R 3.1.2 (R Development Core Team [25]).

## Results

Characteristics of the study population are displayed in Table 1. Mean BMI among the 2270 nurses studied was 26.2 kg/m^2^, and the prevalence of overweight and obesity was 30.8 % and 20 %, respectively. The data set comprised mainly white women (58.2 %), who lived with a partner (57 %), whose mothers had 0 to 8 years’ schooling (49.6 %), whose domestic overload was low (64.5 %), who rated their own health as good (65.2 %), consumed fried food from 1 to 3 times a week (41.4 %) and were physically inactive (69.9 %). With regard to continuous variables, the mean age of the study population was 39.5 years and mean time working nights was 7.1 years. At age 20 years, the mean BMI of the nurses investigated was 21.2 kg/m^2^.

**Table 1:** Characteristics of study population (*n* = 2270)

Variables	n (%) / mean (SD)
Age (years)	39.5 (9.7)
Marital status (%)	
Without partner	977 (43.0)
With partner	1293 (57.0)
Race/colour (%)	
Black	248 (10.9)
Brown	700 (30.8)
White	1322 (58.2)
Mother’s schooling (%)	
0–8 years	1125 (49.6)
9–11 years	734 (32.3)
12 or more years	411 (18.1)
Domestic overload (%)	
Low	1465 (64.5)
High	805 (35.5)
Years worked at night (years)	7.1 (6.9)
Self-rated health (%)	
Good	1480 (65.2)
Poor	790 (34.8)
Consumption of fried food (%)	
Never or less than 1×/month	170 (7.5)
1–3×/month	754 (33.2)
1–3×/week	940 (41.4)
4–6×/week	269 (11.9)
Daily	137 (6.0)
Physical inactivity (%)	
No	683 (30.1)
Yes	1587 (69.9)
BMI at age 20 years (kg/m^2^)	21.2 (3.4)
Current BMI (kg/m^2^)	26.2 (5.1)
Nutritional status (%)	
Normal weight	1117 (49.2)
Overweight	700 (30.8)
Obesity	453 (20.0)

*SD* standard deviation

Table 2 shows the models with BMI in its continuous form as dependent variable. The parameter estimates presented in the table reflect the change in BMI (in kg/m^2^) for each one-unit difference for continuous covariates or switching from one category to the other for categorical covariates. For example, a 1-year increase in age was associated with a 0.15 kg/m^2^ higher BMI in the gamma and linear models, while for quantile models this increase was 0.11, 0.14 and 0.18 kg/m^2^ for the 0.25, 0.50 and 0.75 quantiles, respectively. The models resulting from gamma and linear regressions display the same significant variables and the coefficient values were similar. In the quantile regression, all the variables considered were also significant for the 0.75 quantile. In the models, however, the variable *years worked at night* was not significantly associated with BMI in the 0.25 and 0.50 quantiles, nor was *mother’s schooling* in the latter quantile. Of the models evaluated, the gamma model (AIC = 12,183) returned best fit, followed by the models for the 0.25 and 0.5 quantiles (AIC = 12,310 and AIC = 12,318, respectively). The worst fit was observed in the linear model (AIC = 12,601) and the model for the 0.75 quantile (AIC = 12,954), while residual distribution in the former did not support the normality assumption (see Additional file 2).

**Table 2:** Coefficients and 95 % confidence intervals (CI) of the multivariable linear, gamma and quantile models

Variables	Coefficient (95 % CI)				
	Gamma	Linear	Quantile		
			Quantile 0.25	Quantile 0.50	Quantile 0.75
Age	0.15 (0.14, 0.17)	0.15 (0.13, 0.17)	0.11 (0.09, 0.12)	0.14 (0.12, 0.16)	0.18 (0.16, 0.21)
Marital status^a^					
With partner	0.59 (0.28, 0.90)	0.60 (0.26, 0.93)	0.43 (0.16, 0.79)	0.54 (0.23, 0.85)	0.76 (0.39, 1.14)
Race/colour^b^					
Brown	−0.96 (−1.51, −0.42)	−0.97 (−1.53, −0.41)	−0.43 (−0.98, 0.12)	−0.89 (−1.65, −0.14)	−1.63 (−2.30, −0.96)
White	−1.19 (−1.71, −0.68)	−1.23 (−1.76, −0.70)	−0.73 (−1.21, −0.25)	−1.28 (−2.03, −0.53)	−1.86 (−2.50, −1.22)
Mother’s schooling^c^					
9–11 years	0.21 (−0.13, 0.56)	0.21 (−0.17, 0.58)	−0.10 (−0.40, 0.20)	0.27 (−0.09, 0.62)	0.21 (−0.19, 0.61)
12 or more years	0.69 (0.26, 1.12)	0.80 (0.34, 1.26)	0.42 (0.02, 0.81)	0.34 (−0.09, 0.76)	0.78 (0.13, 1.43)
Domestic overload^d^					
High	0.60 (0.27, 0.94)	0.63 (0.28, 0.98)	0.43 (0.11, 0.74)	0.61 (0.23, 0.99)	0.89 (0.48, 1.31)
Years worked at night	0.03 (0.01, 0.06)	0.03 (0.01, 0.06)	0.00 (−0.03, 0.02)	0.03 (−0.01, 0.06)	0.04 (0.01, 0.07)
Self-rated health^e^					
Poor	1.52 (1.20, 1.85)	1.62 (1.28, 1.97)	1.12 (0.79, 1.45)	1.50 (1.12, 1.87)	1.75 (1.32, 2.19)
Consumption of fried food^f^					
1–3×/month	1.00 (0.41, 1.58)	1.06 (0.42, 1.71)	0.93 (0.26, 1.61)	0.90 (0.25, 1.55)	0.99 (0.02, 1.96)
1–3×/week	1.22 (0.64, 1.80)	1.27 (0.62, 1.91)	0.86 (0.20, 1.52)	0.95 (0.28, 1.63)	1.24 (0.24, 2.24)
4–6×/week	1.41 (0.71, 2.11)	1.61 (0.84, 2.37)	1.02 (0.30, 1.74)	1.20 (0.38, 2.02)	1.55 (0.45, 2.65)
Daily	1.50 (0.69, 2.32)	1.42 (0.53, 2.30)	0.82 (0.13, 1.51)	1.01 (0.17, 1.84)	2.14 (0.99, 3.29)
Physical inactivity^g^					
Yes	0.75 (0.42, 1.80)	0.80 (0.43, 1.15)	0.45 (0.17, 0.74)	0.78 (0.46, 1.10)	0.88 (0.49, 1.27)
BMI at age 20 years	0.93 (0.88, 0.99)	0.90 (0.81, 0.91)	0.84 (0.78, 0.90)	0.97 (0.92, 1.03)	1.09 (1.03, 1.16)

Reference categories: ^a^ “Without partner”; ^b^ “Black”; ^c^ “0–8 years”; ^d^ “Low”; ^e^ “Good”; ^f^ “Never or less than 1×/month”; ^g^ “No”

Similar results were observed in the models where the dependent variable BMI was considered in its categorical form (Table 3). In this table, the results are expressed as OR and indicate the change in overweight and obesity likelihood for each one-unit difference for continuous covariates or switching from one category to the other for categorical covariates. For example, high domestic overload was associated to an increase of 60 % in the likelihood of overweight in the binary logistic model, and of 52 % and 80 % in the likelihood of overweight and obesity, respectively, in the multinomial logistic model. All the variables, except for *years worked at night*, remained significant in both binary and multinomial logistical models. In addition, in the multinomial model, the variables *marital status* and *mother’s schooling* were significantly associated with obesity, but not with overweight.

**Table 3:** Odds ratios (OR) and 95 % confidence intervals (CI) of the multivariable binary and multinomial logistic models

Variable	OR (95 % CI)		
	Binary	Multinomial	
		Overweight	Obesity
Age	1.09 (1.08, 1.11)	1.08 (1.07, 1.10)	1.12 (1.10, 1.15)
Marital status^a^			
With partner	1.32 (1.07, 1.63)	1.23 (0.98, 1.53)	1.64 (1.22, 2.21)
Race/colour^b^			
Brown	0.56 (0.39, 0.82)	0.60 (0.41, 0.88)	0.47 (0.29, 0.76)
White	0.41 (0.28, 0.58)	0.43 (0.30, 0.62)	0.36 (0.23, 0.56)
Mother’s schooling^c^			
9–11 years	1.09 (0.86, 1.38)	1.00 (0.78, 1.28)	1.37 (0.98, 1.91)
12 or more years	1.40 (1.04, 1.89)	1.16 (0.84, 1.59)	2.22 (1.50, 3.30)
Domestic overload^d^			
High	1.60 (1.28, 1.99)	1.52 (1.21, 1.92)	1.80 (1.34, 2.42)
Years worked at night	1.00 (0.98, 1.02)	0.99 (0.97, 1.01)	1.02 (0.99, 1.04)
Self-rated health^e^			
Poor	2.17 (1.74, 2.70)	1.83 (1.45, 2.31)	3.31 (2.47, 4.44)
Consumption of fried food^f^			
1–3×/month	1.96 (1.30, 2.95)	1.81 (1.18, 2.77)	2.44 (1.34, 4.43)
1–3×/week	1.69 (1.13, 2.54)	1.44 (0.94, 2.20)	2.62 (1.45, 4.73)
4–6×/week	2.44 (1.05, 3.96)	2.21 (1.33, 3.66)	3.23 (1.61, 6.45)
Daily	2.05 (1.16, 3.63)	1.90 (1.05, 3.43)	2.49 (1.11, 5.58)
Physical inactivity^g^			
Yes	1.58 (1.26, 1.99)	1.44 (1.13, 1.83)	2.07 (1.49, 2.87)
BMI at age 20 years	1.63 (1.55, 1.71)	1.52 (1.44, 1.60)	1.95 (1.83, 2.07)

Reference categories: ^a^ “Without partner”; ^b^ “Black”; ^c^ “0–8 years”; ^d^ “Low”; ^e^ “Good”; ^f^ “Never or less than 1×/month”; ^g^ “No”

Table 4 summarises the significant variables in each model, as well as the main differences among them. The variables *age*, *marital status*, *race/colour*, *domestic overload*, *self-rated health*, *physical inactivity* and *BMI at age 20 years* were significantly associated with BMI, regardless of the method used. *Consumption of fried food* was significant in all models, but the gradient of the effect varied. A dose–response pattern was identified only in the gamma and normal models and in the quantile regression for the 0.75 quantile. Also, it was only in these three models that *years worked at night* showed a significant effect on BMI. The greatest differences among the models evaluated related to the variable *mother’s schooling*. Significant results were observed only for the 12 or more years of schooling category, except for overweight in the multinomial model and the 0.5 quantile in the quantile model, where the two categories were not significant.

**Table 4:** Significant variables in the models with linear, gamma, quantile, binary and multinomial logistic regression

Variable	Gamma	Linear	Quantile			Binary logistic	Multinomial logistic	
			0.25	0.50	0.75		Overweight	Obesity
Age	+	+	+	+	+	+	+	+
Marital status^a^								
With partner	+	+	+	+	+	+	[+]	+
Race/colour^b^								
Brown	-	-	[-]	-	-	-	-	-
White	--	--	--	--	--	--	--	--
Mother’s schooling^c^								
9–11 years	[+]	[+]	[-]	0	[+]	[+]	[+]	[+]
12 or more years	++	++	+	0	++	++	[++]	++
Domestic overload^d^								
High	+	+	+	+	+	+	+	+
Years worked at night	+	+	0	0	+	0	0	0
Self-rated health^e^								
Poor	+	+	+	+	+	+	+	+
Consumption of fried food^f^								
1–3×/month	+	+	+++	+	+	++	++	+
1–3×/week	++	++	++	++	++	+	[+]	+++
4–6×/week	+++	+++	++++	++++	+++	++++	++++	++++
Daily	++++	++++	+	+++	++++	+++	+++	++
Physical inactivity^g^								
Yes	+	+	+	+	+	+	+	+
BMI at age 20 years	+	+	+	+	+	+	+	+

Reference categories: ^a^ “Without partner”; ^b^ “Black”; ^c^ “0–8 years”; ^d^ “Low”; ^e^ “Good”; ^f^ “Never or less than 1×/month”; ^g^ “No”

The symbols “+” and “-” denote the direction of the association (“+” direct association, and “-” inverse association). The quantity of these symbols indicate the strength of the association (i.e., “++” indicate a stronger association than “+”, and “+++” indicate a stronger association than “++”). The symbol “0” indicate non-significant variables, and the symbol “[]” indicate only non-significant categories

**Fig. 1:**
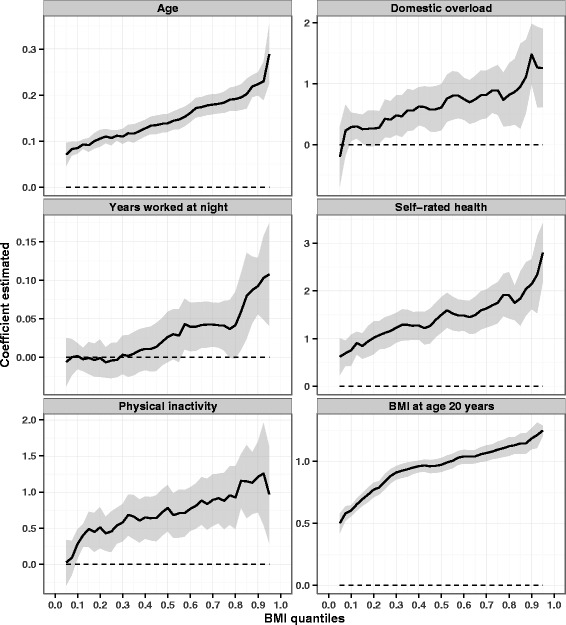
Effects of selected variables on the percentiles of the BMI distribution estimated through multivariate quantile models. On the horizontal axes are the BMI distribution percentiles; the vertical axes show the values of the coefficients estimated. Dashed parallel line represents the null value (zero), and a solid line stands for quantile estimates. A grey area surrounding the solid line represents the 95 % confidence intervals (CI) for the quantile estimates. The explanatory variables evaluated were: age, domestic overload, years worked at night, self-rated health, physical inactivity, and BMI at age 20 years. All coefficients are adjusted by the other study variables

Variables selected for illustration purposes, and their effects on BMI in the gamma and quantile regressions, are plotted in Fig. 1. As can be seen, the independent effect of the variables selected increases the higher the distribution quantile. *Age*, *self-rated health* and *BMI at age 20 years* are significantly associated with BMI in all quantiles. *Domestic overload* and *physical inactivity* start to be significant at approximately the 10th quantile and *years worked at night* at the 55th quantile.

Table 5 compares the estimates from each model for the three profiles of individual described. As expected, the BMI values estimated are lowest for the healthy profile, followed by the intermediate and unhealthy profiles, regardless of the method considered. The BMI values estimated for the healthy and unhealthy profiles by gamma regression were, respectively, slightly lower and a little higher than those estimated by linear regression. For the intermediate profile, however, the values were similar. Note also that the likelihood of overweight in the healthy profile is practically null, but increases to over 50 % in the intermediate profile and reaches nearly 100 % in the unhealthy profile. The likelihood of overweight, in turn, is very low for the extreme profiles, but reaches approximately 35 % among individuals with the intermediate profile. The likelihood of obesity is nearly 16 % for the intermediate profile and exceeds 90 % among the unhealthy.

**Table 5:** Values estimated for each model for the three profiles of individual considered (healthy, intermediate and unhealthy)

Models	Profiles		
	Healthy	Intermediate	Unhealthy
Gamma			
(E[BMI])	16.79	26.13	38.15
Linear			
(E[BMI])	17.02	26.07	37.69
Quantile			
0.25 (E[BMI])	16.59	23.56	32.36
0.50 (E[BMI])	16.69	25.86	37.02
0.75 (E[BMI])	17.06	27.86	43.26
Logistic			
Overweight (P[BMI ≥ 25])	0.7	51.4	99.8
Multinomial			
Normal (P[BMI < 25])	98.8	49.5	0.0
Overweight (P[BMI ≥ 25 e BMI < 30])	1.2	34.7	7.0
Obesity (P[BMI ≥ 30])	0.0	15.8	92.9

E[BMI] = expected value of BMI, in kg/m^2^; P[BMI] = probability of BMI ≥ e/or < certain value, in %

## Discussion

The analyses indicate better fit from the gamma model than the linear regression, especially given the characteristics of the distribution of the response variable, BMI. Quantile regression proved useful as a complementary analytical strategy by making it possible to identify effects present in specific parts of the distribution. Binary and multinomial logistic regression can be good alternatives, depending on the purpose of the study, given that categorisation of a continuous variable entails loss of information.

In modelling variables with characteristics like those of BMI (which is continuous, asymmetrically distributed and strictly positive), the gamma model is a useful approach, because it fits the data well, as shown. In addition, the coefficients are easier to interpret when the identity link function is used. The linear model, meanwhile, did not fit BMI well, as also observed by Fonseca et al. [8], who recommend, when using the linear model, to transform the variable BMI, but that procedure makes results harder to interpret.

With quantile regression, unlike the gamma and linear models, there is no need to assume a distribution for the response variable; the only assumption is that it is continuous. As shown, the results from the quantile regression provide additional information on the effects of the explanatory variables considered in the study. The effects were observed to be greater in the upper quantiles of the distribution. Accordingly, as BMI distribution is right-skewed (mean greater than the median), the coefficients returned by the gamma and linear models, which estimate effects for the mean, are generally larger than those observed for the 0.25 and 0.5 (median) quantiles in the quantile model and lower than estimates for the 0.75 quantile.

According to AIC, the fit for the 0.75 quantile regression model was worse than the fit of the models for lower quantiles and of the gamma regression model. Although quantile regression has not any reliance on global distributional assumptions, as we have emphasized, the estimation process is influenced by the local features of the distribution near the specified quantile [23]. Thus, the worst fit for the 0.75 quantile might be related to the asymmetric behaviour of the BMI leading to higher heterogeneity of the people with BMI around the theoretical 0.75 quantile. More research is needed to tackle this issue.

When modelling with the response variable categorised, in the multinomial model, the effects were always greater for obesity (BMI ≥ 30) than for overweight (25 ≤ BMI < 30). In the binary logistic regression, overweight and obese individuals are considered in a single category and therefore the values estimated are intermediate in relation to those found for the categories separately. The agreement observed between the logistic (binary and multinomial) models and the quantile model for the 0.75 quantile stems from the fact that these models rest on similar bases. The advantage of the quantile model over the others is that there is no information loss resulting from categorisation of the response variable [26]. In that respect, the results showed that the variable *years worked at night* was significant in the quantile model for the 0.75 quantile, but not in the binary and multinomial logistic models, suggesting that information loss resulting from categorisation of the response variable in the latter two models made it impossible to observe an association that exists only in the upper part of the distribution.

Other techniques may be used in the context of studies of determinants of overweight, but these lay beyond the scope of this study, given that the main objective was to compare the strategies most used in the literature to model BMI data. As the focus of the article was to compare models on the basis of the same data set, limitations relating to data acquisition and treatment do not affect the conclusions. In addition, the sample treatment is a strong point of the study.

## Conclusions

The models evaluated returned similar results for the explanatory variables significantly associated with BMI in both its continuous and categorical forms, even though the magnitude of the associations did vary among the models. The modelling strategies evaluated differed mainly in terms of goodness of fit and the ease of interpretation of the parameters, and in most instances they can be used complementarily. Accordingly, the choice of model to be used should always consider the nature of the data and, most importantly, the purpose of the study.

## Abbreviations

AIC, Akaike Information Criterion; BMI, body mass index; CI, confidence interval; OR, odds ratio; SD, standard deviation

## Additional files

Additional file 1: Questionnaire. Description of data: The self-completed questionnaire, covering characteristics of professional and household work, health and socioeconomic. (PDF 255 kb) Additional file 2: Analysis of standardized Pearson residuals from the linear model. Description of data: This additional file presents the analysis of standardized Pearson residuals from the linear model, demonstrating that the distribution of the residuals did not support the normality assumption. (PDF 87 kb)
