# Supplementary material for: Factors associated with overweight: are the conclusions influenced by choice of the regression method?
Source: BMC Public Health. 2016 Jul 26;16:642. doi: 10.1186/s12889-016-3340-2 (PMC4962412; doi:10.1186/s12889-016-3340-2)
Supplement: Additional file 1: — Questionnaire. Description of data: The self-completed questionnaire, covering characteristics of professional and household work, health and socioeconomic. (PDF 255 kb) [file 12889_2016_3340_MOESM1_ESM.pdf]

Additional file 1 - Questionnaire

I`LL BEGIN THIS INTERVIEW ASKING A FEW QUESTIONS ABOUT YOUR WORK

A1. In how many places do you work on nursing?

1 □ One
2 □ Two
3 □ Three or more

A2. Do you work regularly (at least once a week / 4 times a month) at night shifts in nursing assistance in some place?

1 □ Yes
2 □ No **GO TO A4**

A3. For how long have you been working at night in nursing, here or in another place?

________________years. □ Less than one year. **GO TO B1**

A4. Have you ever worked nights?

1 □ Yes, regularly, at least once a week.
2 □ Yes, occasionally or rarely.
3 □ No **GO TO B1**

A5. For how long did you work at night?

________________years. □ Less than one year.

I WOULD NOW LIKE TO ASK SOME QUESTIONS ABOUT YOUR HEALTH

B1. In a general way, compared to people you age, how do you consider your health to be?

1 □ Very good	2 □ Good	3 □ Regular	4 □ Bad	5 □ Very bad

B2. In relation to your sleep, how often did you:

	**Never**	**Rarely**	**A few times**	**Almost always**	**Always**
**a)** Have difficulty falling asleep?	1 □	2 □	3 □	4 □	5 □
**b)** Waking up during the night (more than three times)?	1 □	2 □	3 □	4 □	5 □
**c)** Wake up before the desired time and not managing to sleep again?	1 □	2 □	3 □	4 □	5 □

B3. How much you weighed at age 20, approximately?

___ ___ ___. ___ ___ ___ Kg 777 □ don't know/don't remember

B4. What is your current weight (if pregnant, tell your pre-pregnancy weight)?

___ ___ ___. ___ ___ ___ Kg 777 □ don't know/don't remember

B5. What is your height?

___ ___ ___ cm   777 ☐ don't know/don't remember

B6. In the last two weeks, have you done any kind of physical activity?

1 ☐ Yes
2 ☐ No

B7. Do you or did you ever take pills or hormonal contraceptives to avoid pregnancy?

1 ☐ Yes. If yes, for how long?
→ 1 ☐ Less than a year   2 ☐ Between 1 and 5 years   1 ☐ More than 5 years
2 ☐ No

B8. Do you smoke?

1 ☐ Yes . In general, how many cigarettes do you smoke a day?
→ ____________ cigarettes.
2 ☐ No. I smoked in the past, but I quit.
3 ☐ No. I've never smoked.

B9. How often do you consume alcoholic drink?

1 ☐ Never
2 ☐ Once a month or less
3 ☐ 2 to 4 times a month
4 ☐ 2 to 3 times a week
5 ☐ 4 or more times a week

B10. How often do you eat fried food (deep-fried)?

1 ☐ Never or less than once a month
2 ☐ 1 to 3 times a month
3 ☐ 1 to 3 times a week
4 ☐ 4 to 6 times a week
5 ☐ Daily

B11. How often do you eat fresh fruit?

1 ☐ Never or less than once a month
2 ☐ 1 to 3 times a month
3 ☐ 1 to 3 times a week
4 ☐ 4 to 6 times a week
5 ☐ Daily

B12. How often do you eat vegetables?

1 ☐ Never or less than once a month
2 ☐ 1 to 3 times a month
3 ☐ 1 to 3 times a week
4 ☐ 4 to 6 times a week
5 ☐ Daily

THE NEXT QUESTIONS ARE ABOUT ASPECTS OF YOU PERSONAL HISTORY

C1. What is the educational background of your mother?

1 □ didn't go to school
2 □ Elementary school incomplete
3 □ Elementary school complete
4 □ Secondary school incomplete
5 □ Secondary school complete
6 □ University (College) incomplete
7 □ University (College) complete
8 □ didn't know her/died early

C2. What is your age? ________________ years

C3. What is your educational background?

1 □ University degree complete
3 □ Master degree
2 □ Post-graduation (specialization, residency)
4 □ Doctorate degree

C4. What is your marital status?

1 □ married or living together
2 □ separated or divorced
3 □ widow(er)
4 □ single (Never been married or lived in a union)

C5. How many children born alive do you have or did you have?

________________ children
0 □ I did not have children.

C6. Do you have children under six years old living with you?

1 □ Yes
2 □ No

C7. The Brazilian Institute of Statistics (IBGE) uses the words black, brown, white, yellow and indigenous to classify people's colour or race. If you had to answer the IBGE survey today, how would you classify yourself in this respect?

1 □ Black	2 □ Brown	3 □ White	4 □ Yellow	5 □ Indigenous

C8. Including you, how many people live in your house? ________________ people

C9. When you are at home, is it your responsibility to:

**Task**	**Yes, entirely**	**Yes, most of the time**	**Divided equally**	**Yes, partly**	**No**
Take care of children/teenagers?<br>9 □ I don't have children.	1 □	2 □	3 □	4 □	5 □
Do the cleaning?	1 □	2 □	3 □	4 □	5 □
Cook?	1 □	2 □	3 □	4 □	5 □
Wash clothes?	1 □	2 □	3 □	4 □	5 □
Iron clothes?	1 □	2 □	3 □	4 □	5 □
