# Supplementary figures and images for: Factors associated with overweight: are the conclusions influenced by choice of the regression method?
Source: BMC Public Health. 2016 Jul 26;16:642. doi: 10.1186/s12889-016-3340-2 (PMC4962412; doi:10.1186/s12889-016-3340-2)

**Additional file 2 - Analysis of standardized Pearson residuals from the linear model**

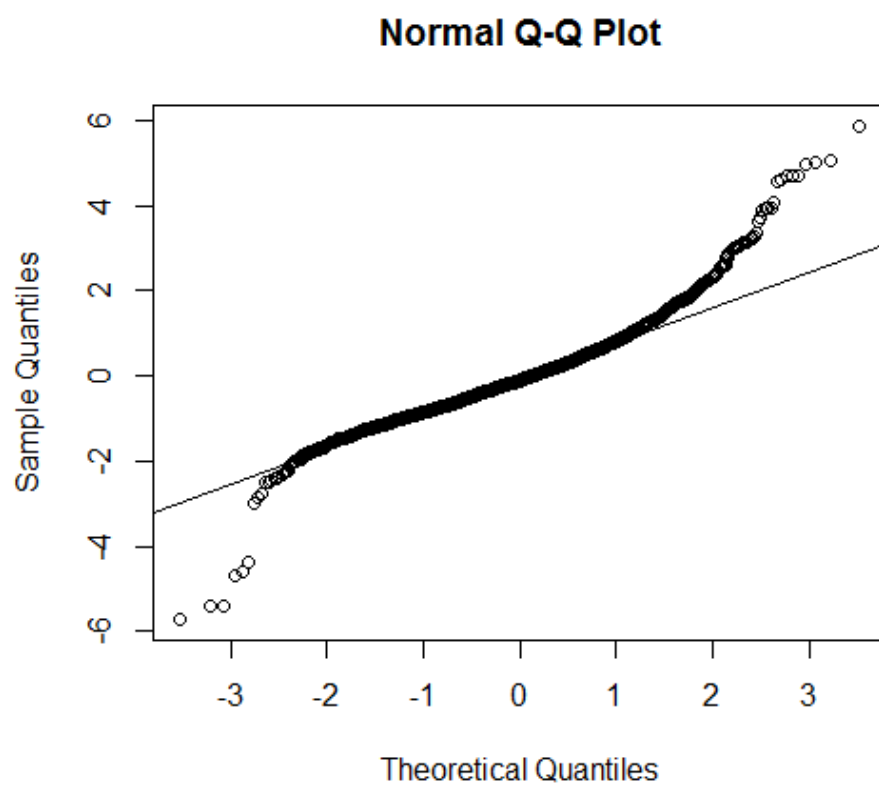

Supplement: Additional file 2: — Analysis of standardized Pearson residuals from the linear model. Description of data: This additional file presents the analysis of standardized Pearson residuals from the linear model, demonstrating that the distribution of the residuals did not support the normality assumption. (PDF 87 kb) [file 12889_2016_3340_MOESM2_ESM.pdf]
